# A real-world pharmacovigilance assessment and literature review of lymphoma development in lipodystrophy

**DOI:** 10.3389/fendo.2025.1582715

**Published:** 2025-05-21

**Authors:** Rebecca J. Brown, David Araujo-Vilar, Kelly J. Walkovich, Alexandru Barbarosie, David A. Magee, Baris Akinci, Elif A. Oral

**Affiliations:** ^1^ Diabetes, Endocrinology, and Obesity Branch, National Institute of Diabetes and Digestive and Kidney Diseases, National Institutes of Health, Bethesda, MD, United States; ^2^ UETeM−Molecular Pathology of Rare Diseases Group, Center for Research in Molecular Medicine and Chronic Diseases (CIMUS), School of Medicine and Dentistry, University of Santiago de Compostela, Santiago de Compostela, Spain; ^3^ Division of Pediatric Hematology/Oncology, Department of Pediatrics, University of Michigan, Ann Arbor, MI, United States; ^4^ Chiesi Global Rare Diseases, Dublin, Ireland; ^5^ Izmir Biomedicine and Genome Center & Dokuz Eylul University Technopark (DEPARK), Dokuz Eylul University Health Campus, Izmir, Türkiye; ^6^ Metabolism, Endocrinology and Diabetes Division, Department of Internal Medicine, Caswell Diabetes Institute, North Campus Research Complex, University of Michigan, Ann Arbor, MI, United States

**Keywords:** acquired generalized lipodystrophy, T-cell lymphoma, autoimmunity, B cell lymphoma, lymphoma, lipodystrophy, metreleptin, pharmacovigilance

## Abstract

**Introduction:**

Metreleptin is a form of leptin replacement therapy used with diet and lifestyle modifications to treat the metabolic complications of leptin deficiency in lipodystrophy, a rare disease characterized by adipose tissue deficiency. Previously, identification of T-cell lymphomas in three metreleptin-treated patients with acquired generalized lipodystrophy (AGL) suggested a possible relationship between metreleptin and lymphoma development. To further investigate this, we performed a real-world pharmacovigilance assessment and literature review to identify lymphomas in patients with lipodystrophy and congenital leptin deficiency (CLD) who were either metreleptin-naïve, or who had previously received/were receiving metreleptin at the time of lymphoma diagnosis.

**Methods:**

Cases were identified from PubMed, Embase and the Cochrane Library (from database inception through to November 22, 2024), and through review of 11 years post-marketing data from the global safety database (GSD) of the marketing authorization holder for metreleptin.

**Results:**

The final analysis set comprised 17 lymphomas in 16 patients reported in 11 published articles and one GSD case report. Twelve lymphomas were recorded in 12 metreleptin-naïve patients — these comprised six T-cell lymphomas (one each in six patients with AGL), three B-cell lymphomas (in two patients with familial partial lipodystrophy and one patient with AGL), and three Hodgkin lymphomas (separately reported in one patient each with generalized lipodystrophy, juvenile-onset dermatomyositis-associated lipodystrophy, and CLD). Five lymphomas were identified in four metreleptin-treated patients, three of whom (all with AGL and T-cell lymphomas) were reported in previously published studies. The remaining metreleptin-treated patient (from the GSD) had generalized lipodystrophy-associated atypical progeroid syndrome and developed a B-cell lymphoma and brain lymphoma following solid organ transplantation and immunosuppressant therapy. All nine T-cell lymphomas occurred in patients with AGL, and additional autoimmune and/or inflammatory disorders were commonly reported in these patients.

**Discussion:**

While a contributory role for metreleptin in lymphoma development in patients with lipodystrophy cannot be excluded, our analysis suggests that lymphoma development may be associated with underlying pathophysiology that also leads to lipodystrophy rather than the pharmacological actions of metreleptin. Our findings support the view that, in some instances, immunoproliferative disorders of T-cells may contribute to syndromes involving autoimmune processes, including AGL.

## 1 Introduction

Lipodystrophy syndromes are a heterogenous group of rare diseases characterized by a lack of adipose tissue affecting either the whole body (i.e., generalized lipodystrophy, GL) or specific areas (partial lipodystrophy, PL) (1, 2). The etiology of GL and PL may be genetic or acquired resulting in four main lipodystrophy types: congenital generalized lipodystrophy (CGL, or Berardinelli–Seip syndrome), familial partial lipodystrophy (FPLD, including Köbberling syndrome and Dunnigan variety), acquired generalized lipodystrophy (AGL, or Lawrence syndrome), and acquired partial lipodystrophy (APL, or Barraquer–Simons syndrome) (1–3). Genetic lipodystrophy syndromes are generally associated with impaired adipocyte function and development while acquired lipodystrophy syndromes are thought to arise from autoimmune or inflammation-mediated destruction of adipocytes (4–8).

Deficiency of adipose tissue in lipodystrophy syndromes results in ectopic lipid deposition and is frequently associated with reduced levels of leptin, a key adipokine regulator of appetite and energy homeostasis (1, 2, 9, 10). Consequently, patients are predisposed to the development of severe metabolic and organ system comorbidities including severe insulin resistance, hypertriglyceridemia, steatotic liver disease, pancreatitis, and renal and cardiovascular disease (1, 11–13). Absolute deficiency of leptin is also a clinical feature of congenital leptin deficiency (CLD), an ultrarare genetic disorder associated with biallelic pathogenic variants in the leptin gene and characterized by extreme hyperphagia and several neuroendocrine defects (14–17). Unlike lipodystrophy syndromes, reduced adiposity is not a feature of CLD; instead, surplus calories are deposited in functioning adipose tissue causing severe and refractory early-onset obesity (17).

The clinical program for metreleptin, a recombinant form of human leptin, was initiated in 2000 in the US, with outcomes reported for the first nine treated patients in 2002 (18–20). The efficacy and safety findings from this clinical program led to subsequent approvals for metreleptin in several regions and countries. Metreleptin was first approved in Japan in 2013 for the treatment of lipodystrophy (21, 22). In the United States (US), metreleptin is approved (since 2014) as an adjunct to diet as replacement therapy to treat the complications of leptin deficiency in patients with GL (23). Metreleptin is also indicated in Europe (since 2018), and in Brazil and Canada (since 2023 and 2024, respectively) as an adjunct to diet as replacement therapy to treat the complications of leptin deficiency in patients aged ≥ 2 years with confirmed GL, or in patients aged ≥ 12 years with confirmed PL, for whom standard treatments have not achieved adequate metabolic control (24–27). Metreleptin has also been used off-label to treat the metabolic complications of CLD in a limited number of patients (14, 28, 29).

Clinical studies in lipodystrophy show that metreleptin is generally well-tolerated with decreased weight, abdominal pain, hypoglycemia, and nausea among the most commonly reported adverse events (19, 20). The prescribing information for metreleptin in the US and Europe also contains a warning for the risk of anti-metreleptin antibodies and the development of T-cell lymphomas (23–25). However, reports of T-cell lymphomas in metreleptin-naïve patients with AGL have led to suggestions that autoimmune mechanisms leading to the development of AGL may also contribute to lymphoma risk in AGL (30).

To further explore the possible relationship between metreleptin and lymphoma development, we conducted a real-world pharmacovigilance assessment of the global safety database (GSD) of the marketing authorization holder (MAH) for metreleptin, and a literature search of three databases to identify patients with lipodystrophy or CLD who (1): had previously received or were receiving metreleptin at the time of lymphoma diagnosis, or (2) had never received metreleptin up to the time of lymphoma diagnosis. We present our findings based on lipodystrophy type and metreleptin treatment status. Potential contributory mechanisms to lymphoma development in lipodystrophy and CLD are also discussed, with a focus on AGL.

## 2 Methods

### 2.1 Publicly accessible database searches

Searches of PubMed, Embase and the Cochrane Library were conducted from the time of inception of each database up until November 22, 2024. The search strings employed for each database are presented in **Table 1**. For all database searches, information from available published clinical and real-world studies (e.g., manuscripts, case series, case studies, abstracts, congress proceedings, and congress presentations) reporting non-human immunodeficiency virus (HIV)-related fat loss consistent with descriptions of rare lipodystrophy syndromes or lipoatrophy were eligible for inclusion in the analysis. Reports of patients with progeroid syndromes associated with lipodystrophy or CLD were also considered. Publications could be in any language and patients could be of any age. Patients with localized lipodystrophy or lipoatrophy including those secondary to the administration of medications (e.g., steroids, growth hormone, insulin, antibiotics, vaccines), or fat loss due to malnutrition or anorexia nervosa were excluded from the analysis.

**Table 1:** Database search terms.

Database	Search Terms
PubMed	Search 1: lipodystrophy and lymphoma *(((((((((lipodystrophy AND lymphoma) OR (lipoatrophy AND lymphoma)) OR (recombinant leptin AND lymphoma)) OR (metreleptin AND lymphoma)) OR (metreleptin AND cancer)) OR (leptin replacement AND lymphoma)) OR (leptin replacement AND cancer)) NOT (mouse)) NOT (rat)) NOT (review[Publication Type])* Search 2: CLD and lymphoma *((congenital leptin deficiency) AND (lymphoma)) OR ((leptin deficiency AND lymphoma))*
Embase	Search 1: lipodystrophy and lymphoma *(lipodystrophy AND lymphoma OR (lipodystrophy AND cancer) OR (metreleptin AND lymphoma) OR (metreleptin AND cancer) OR (leptin AND replacement AND lymphoma)) NOT (whipples AND disease) NOT rat NOT mouse NOT (in AND vitro AND study) NOT review NOT (systematic AND review) NOT (human AND immunodeficiency AND virus)* Search 2: CLD and lymphoma *(‘congenital leptin deficiency’ AND lymphoma)*
Cochrane Library	*(lipodystrophy OR congenital leptin deficiency OR leptin deficiency) AND (lymphoma OR cancer)*

CLD, congenital leptin deficiency.

### 2.2 Interrogation of the GSD of the MAH for metreleptin

As an additional step, a search of the GSD of the MAH for metreleptin was conducted to identify other relevant data not captured in the database searches. The GSD is a comprehensive pharmacovigilance system designed to manage the collection, monitoring, and reporting of adverse drug reactions and other pharmacovigilance data. This database contains all adverse events reported from spontaneous and solicited sources in patients receiving metreleptin since the first approval of metreleptin in Japan in 2013. The database has been managed by several MAHs, with Amryt Pharmaceuticals DAC (a wholly owned subsidiary of Chiesi Farmaceutici S.p.A) being the MAH responsible for its maintenance at the time of writing. The standardized Medical Dictionary for Regulatory Activities (MedDRA) query (SMQ) “*malignant lymphomas*” was used to search this database for relevant case reports related to lymphoma-related events in patients receiving metreleptin up to November 22, 2024 (31).

### 2.3 Retrospective review of medical history for cases recorded in the GSD of the MAH for metreleptin

A retrospective search of the medical history of patients in the GSD cumulatively up to October 2024 was also performed. This search focused on cases reporting any adverse events/special situations associated with metreleptin to identify any metreleptin-naïve patients with a pre-existing diagnosis of lymphoma reported in their medical history. Terms included in the “*malignant lymphomas*” SMQ were used to search the GSD for cases with lymphoma-related medical histories.

### 2.4 Data refinement and final analysis set

Two authors (DM and BA) independently evaluated the eligibility of identified publications for inclusion in the final analysis set (FAS) by initial screening of title and abstract, followed by full text review of selected publications. For inclusion in the final analysis set, publications were required to describe patients with rare lipodystrophy syndromes or CLD who developed lymphomas and who had either previously received or were receiving metreleptin at the time of lymphoma diagnosis (i.e., metreleptin-treated) or who had never received metreleptin up to the time of lymphoma diagnosis (i.e., metreleptin-naïve). Patients who had never received metreleptin up to the time of lymphoma diagnosis but later received metreleptin were considered metreleptin-naïve in our analysis. Publications without original data (such as reviews, editorials, and commentaries), and publications that described patients with lipodystrophy who had an occurrence of leukemia or non-lymphoma cancer types were not included in the FAS.

Finally, a thorough review of the extracted data was performed to identify duplicate cases. This involved a review of (1): the names of the publication author(s) (2); the name and location of the institution at which the patient(s) had been enrolled (3); the age of the patient(s) at the time of the diagnoses of lipodystrophy, CLD and lymphoma; and (4) a code identifier of each patient, if available. Patients with lipodystrophy or CLD with lymphoma who were described more than once in the publication list (from the database searches) or who were included in the GSD and were previously published were included only once in the FAS. The refinement criteria are listed in **Table 2**.

**Table 2:** PICOS table of inclusion criteria for the final analysis.

Criteria	Inclusion Criteria
Population	Patients diagnosed with rare lipodystrophy syndromes (i.e., AGL, CGL, FPLD and APL) and congenital leptin deficiency with the occurrence of lymphoma
Intervention/ comparator	Metreleptin
Outcomes	Development of lymphoma
Study design	Publications with original human data (e.g., clinical studies, case studies, case series, conference abstracts, conference posters)
Other	English and non-English articles provided an English version of the abstract was available, any geographical region

AGL, acquired generalized lipodystrophy; APL, acquired partial lipodystrophy; CGL, congenital generalized lipodystrophy; FPLD, familial partial lipodystrophy; PICOS, population, intervention, comparator, outcome, and study design.

## 3 Results

### 3.1 Lymphoma and lipodystrophy types in the overall FAS

**Figure 1:**
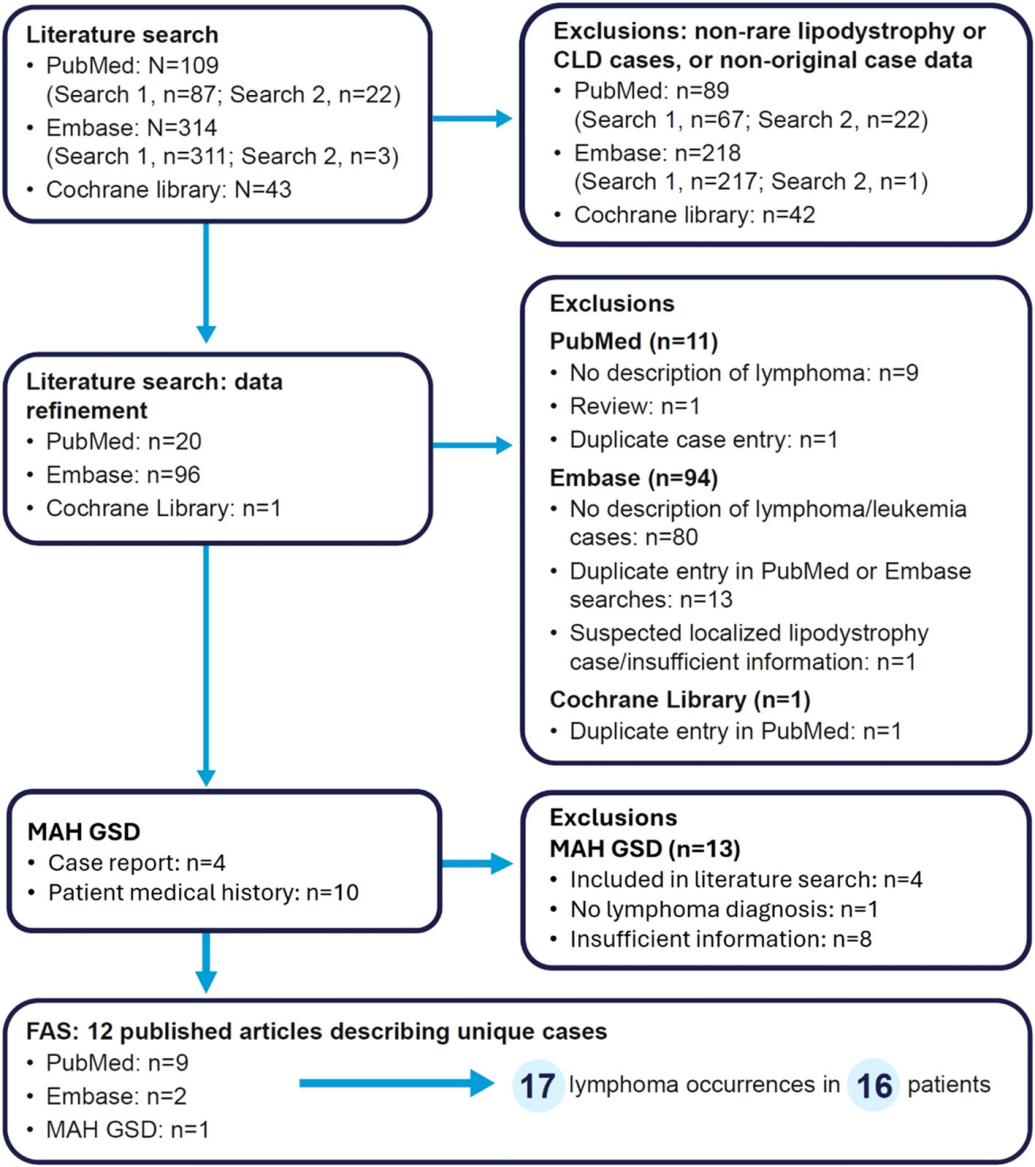
Flow diagram of article/case screening. Search 1 refers to lipodystrophy and lymphoma, while search 2 refers to CLD and lymphoma. For the MAH GSD search, four patients (three with AGL and one with CLD) were identified from the literature search. GSD, global safety database; MAH, marketing authorization holder.

The outcomes of each step of the literature and GSD searches are summarized in **Figure 1**. In total, 11 publications describing unique patients with lipodystrophy or CLD were identified from the literature search (nine from PubMed, two from Embase and none from the Cochrane Library) and one case report was retrieved from the GSD search.

The overall FAS consisted of 17 lymphomas identified in 16 patients (six males, 10 females), of whom 15 had lipodystrophy and one had CLD. Assessment of the group with lipodystrophy showed that 10 patients had AGL, two patients had FPLD, and one patient each had GL, juvenile-onset dermatomyositis (JDM)-associated lipodystrophy, and GL-associated atypical progeroid syndrome (APS). The onset of the lipodystrophy phenotype occurred before the age of 18 years in six of the 15 patients with lipodystrophy. Hyperphagia and weight gain were apparent at age 3 months in the single patient with CLD.

T-cell lymphomas (n=9/17; 53%) were the most frequently reported lymphoma type in the overall FAS, followed by B-cell lymphomas (n=7/17, 41%; including three Hodgkin lymphomas) and a single brain lymphoma (n=1/17, 6%). Notably, all nine T-cell lymphomas were reported exclusively in nine patients with AGL.

### 3.2 Distribution of lymphomas based on metreleptin treatment status in the FAS

**Figure 2:**
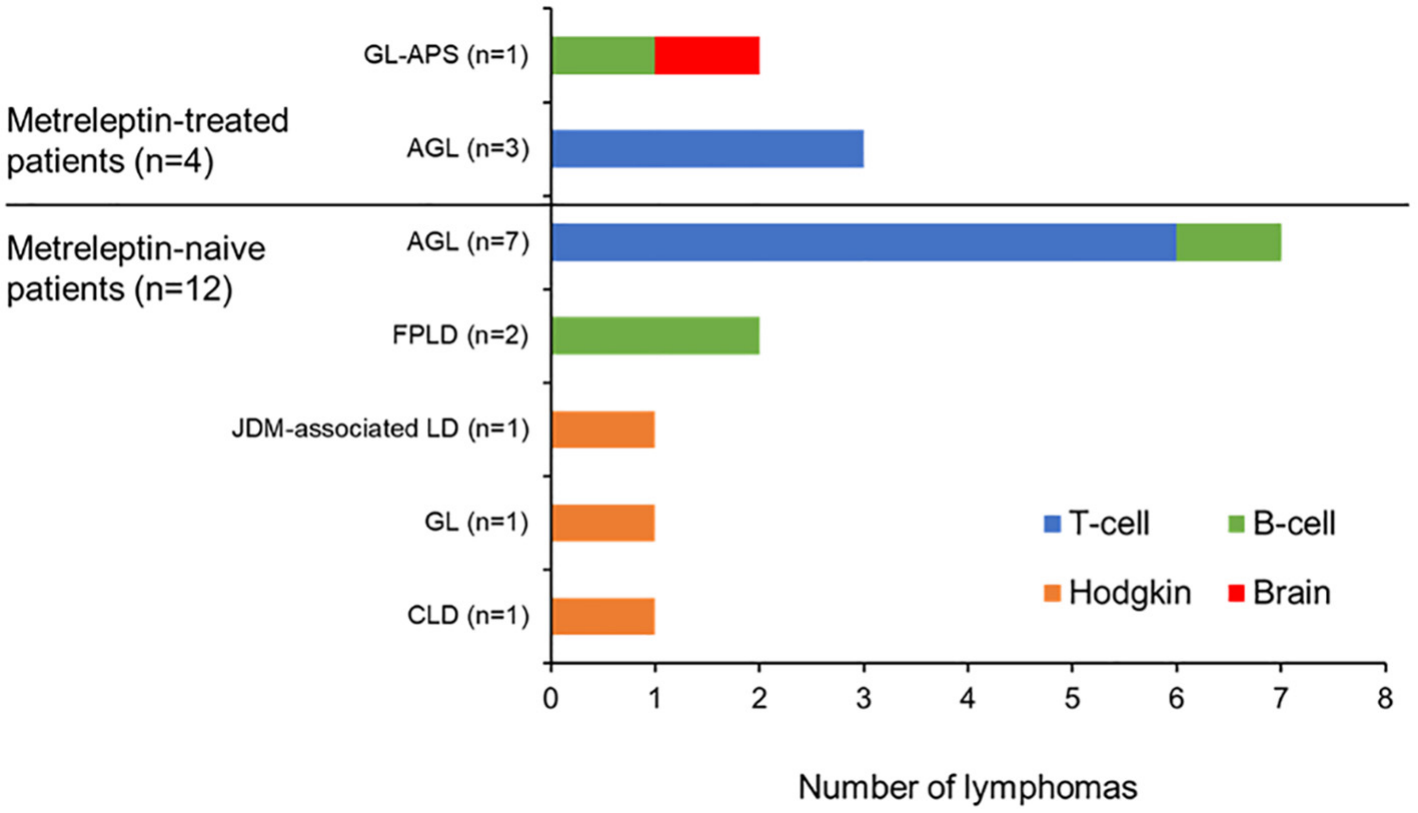
Distribution of lymphoma type across the patients in the FAS. AGL, acquired generalized lipodystrophy; CLD, congenital leptin deficiency; FAS, final analysis set; FPLD, familial partial lipodystrophy; GL, generalized lipodystrophy; GL-APS, GL-associated atypical progeroid syndrome; JDM, juvenile-onset dermatomyositis; LD, lipodystrophy.

The distribution of lymphomas in the FAS according to the metreleptin treatment status of the patients is summarized in **Figure 2**. A synopsis of each case is provided in the **Supplementary Material**. Overall, we identified 16 lymphomas in 15 patients with lipodystrophy syndromes and one lymphoma in a single patient with CLD. Notably, there was a greater number of lymphomas in metreleptin-naïve patients (n=12/16, 75% of patients; n=12/17, 71% of lymphomas) relative to metreleptin-treated patients (n=4/16, 25% of patients; n=5/17, 29% of lymphomas).

#### 3.2.1 Lymphomas in metreleptin-treated patients

Five of the 17 lymphomas (29%) were reported in four metreleptin-treated patients (Patients 1–4, **Table 3**). Two of these lymphomas, considered to be related to one another (one B-cell and one brain lymphoma), occurred in the single patient with GL-associated APS identified from the GSD (Patient 1). The remaining three lymphomas (all T-cell lymphomas) were identified in three patients with AGL who were previously documented in published clinical studies (Patients 2–4).

**Table 3:** Clinical characteristics of metreleptin-treated patients the final analysis set.

Patient Number	Source	Sex	Lipodystrophy type	Approximate age at onset of lipodystrophy (years)	Age at lymphoma diagnosis (years)	Lymphoma type	Other clinical features/notes	Metreleptin treated at lymphoma diagnosis?
1	GSD of MAH for metreleptin [case initially reported prior to the onset of lymphoma (32, 33)]	Male	GL-associated progeroid syndrome	3.5	19 21	Diffuse large B-cell lymphoma Brain lymphoma	• Hypertriglyceridemia • Diabetes • Non-alcoholic steatohepatitis • Severe dilated cardiomyopathy • Metreleptin initiated at age 8 years. • Entered final stages of cardiac function (at age 12 years) necessitating a heart transplant (age 13 years) • Received immunosuppressive pharmacotherapy after heart transplant • EBV infection as complication of heart transplant (~aged 19 years)	Yes (for ~13 years)
2	Brown et al., 2016 (30)	Female	AGL	1–2	13	T-cell lymphoma (ALK+ anaplastic large cell lymphoma)	• Hypertriglyceridemia • Insulin resistance • Metabolic dysfunction-associated steatohepatitis • Metreleptin initiated at ~ 11 years of age • Metreleptin ceased upon lymphoma diagnosis, but re-started 6 weeks later • No history of autoimmunity	Yes (for ~2 years)
3	Brown et al., 2016 (30)	Female	AGL	~49	59	Peripheral T-cell lymphoma	• Hypertriglyceridemia • Diabetes • Hepatic steatosis • GCSF treatment for neutropenia • Metreleptin initiated at 59 years of age	Yes (for <1 year)
4	Brown et al., 2016 (30)	Male	AGL	63	68	Peripheral T-cell lymphoma	• Hypertriglyceridemia • Extreme insulin resistance • Hepatosplenomegaly with steatohepatitis • Leukopenia/neutropenia • Metreleptin initiated at ~67 years of age • Skin lesions observed prior to metreleptin initiation	Yes (for <1 year)
5	Brown et al., 2016 (30)	Female	AGL	60	39	Peripheral T-cell lymphoma (mycosis fungoides)	• AGL diagnosed 25 years after lymphoma diagnosis • AGL developed after a flu-like illness • Hypertriglyceridemia • Diabetes • Anemia • Metreleptin initiated after lymphoma diagnosis (at age 68 years)	No
6	Yiannias et al., 2006 (55)	Male	AGL	Between 32-40	45	Peripheral T-cell lymphoma	• Lymphohistiocytic panniculitis age ~32 years • Hypertriglyceridemia • Leukopenia • Elevated liver transaminases • Autoantibodies	No
7	Aslam et al., 2015 (56)	Male	AGL	Between 47-48	46	Peripheral T-cell lymphoma	• Trisomy 21 • Hepatomegaly • Hypertriglyceridemia • Diabetes • Severe insulin resistance • Suspected autoimmunity	No
8	Esfandiari et al., 2019 (57)	Female	AGL	Before the age of 26 (before the diagnosis of T-cell lymphoma)	Between 26-27	SPTCL	• Stage IV SPTCL with HLH • Allogeneic stem cell transplant for treatment of SPTCL including immunosuppression • AGL present before lymphoma development based on the patient’s medical history and photographs • Hypertriglyceridemia • Diabetes	No
9	Hoff et al., 2024 (42)	Male	AGL	46	34 (spontaneous remission) and possible recurrence at age 46 years	SPTCL	• AGL diagnosed after lymphoma development • Allogenic stem cell transplant for treatment of relapsed SPTCL vs lupus panniculitis with HLH including immunosuppression • Cytopenia • Hypertriglyceridemia	No
10	Ebihara et al., 2018 (58)	Female	AGL	64	58	AITL	• Chemotherapy received for AITL • Diabetes developed after onset of adipose tissue loss • Metreleptin initiated after lymphoma diagnosis (at age 68 years)	No
11	Brown et al., 2016 (30)	Male	AGL	~10	16	B-cell lymphoma (Burkitt lymphoma)	• Hypertriglyceridemia • Diabetes • Acanthosis nigricans • Hepatomegaly • Splenomegaly • Inflammatory disease • Anemia	No
12	de Andrade et al., 2020 (59)	Female	FPLD	~13	40	B-cell follicular lymphoma	• Hypertriglyceridemia • Pancreatitis • Hepatic steatosis • Diabetes • Polycystic ovarian syndrome	No
13	Iwanishi et al., 2014 (60)	Female	FPLD	12	48	Intestinal follicular lymphoma, B-cell	• Diabetes • Atherosclerosis • No history of autoimmunity	No
14	Rego de Figueiredo et al., 2018 (61)	Female	JDM-associated lipodystrophy	38	31	Hodgkin lymphoma	• Immunosuppressive therapy from age 3 to 21 years • Chemotherapy for Hodgkin lymphoma • Generalized calcinosis • Diffuse alopecia • Poikiloderma • Autoantibodies	No
15	Hall et al., 1978 (62)	Female	GL (unclear if acquired vs. genetic)	0.25	22	Hodgkin lymphoma	• Scleroderma • Hemolytic anemia	No
16	Torchen et al., 2021 (63, 64)	Female	CLD	N/A	20	Hodgkin lymphoma	• Hyperphagia • Developmental delay • Hyponatremia • Autoimmune thyroid disease • Growth hormone deficiency • Prediabetes • Metreleptin treatment initiated after remission of lymphoma	No

AGL, acquired generalized lipodystrophy; AITL, angio-immunoblastic T-cell lymphoma; CLD, congenital leptin deficiency; FPLD, familial partial lipodystrophy; GCSF, granulocyte colony stimulating factor; GL, generalized lipodystrophy; HLH, hemophagocytic lymphohistiocytosis; JDM, juvenile-onset dermatomyositis; SPTCL, subcutaneous panniculitis-like T-cell lymphoma.

Patient 1 was first documented in a real-world metreleptin study published in 2015 (32) and was later confirmed as being heterozygous for the *LMNA* c.29C>T (p.T10I) variant (33). Lymphomas developed in this patient after these earlier publications and are described here for the first time. In brief, metreleptin was initiated in Patient 1 at age 8 years following the onset of severe metabolic complications and dilated cardiomyopathy. Due to worsening heart failure and limited cardiac function (at age 12 years), the patient underwent heart transplantation and received immunosuppressant therapy (32). A post-transplant B-cell lymphoma with Epstein–Barr virus (EBV) positivity was reported (at age ~19 years) in the large intestine, necessitating chemotherapy. Approximately 2 years later, the patient developed a brain lymphoma that was considered related to the prior B-cell lymphoma. Lymphoma development in this patient was considered not related to metreleptin treatment by the treating physician.

#### 3.2.2 Lymphomas in metreleptin-naive patients

Twelve of the 17 lymphomas (71%) in the FAS occurred in 12 metreleptin-naïve patients (11 patients with lipodystrophy and one patient with CLD) (Patients 5–16, **Table 3**). Seven of these lymphomas (six T-cell lymphomas and one B-cell lymphoma) were identified in seven patients with AGL (Patients 5–11), two lymphomas (both B-cell lymphomas) occurred in the two patients with FPLD (Patients 12 and 13), and a Hodgkin lymphoma was reported in each of the single patients with JDM-associated lipodystrophy (Patient 14), GL (Patient 15; possibly a patient with CGL due to a generalized lack of fat evident from the age of 3 months), and CLD (Patient 16).

Further inspection of the 11 metreleptin-naïve patients with lipodystrophy revealed that six of these individuals developed lymphoma after the onset of adipose tissue loss (Patients 6, 8, 11–13, and 15), while lymphomas developed before or near to the time of onset of adipose tissue loss in the other five patients (Patients 5, 7, 9, 10 and 14).

### 3.3 Retrospective review of medical history for cases recorded in the GSD of the MAH for metreleptin

A retrospective search of the GSD identified a total of 10 patients who had a *“malignant lymphoma”* SMQ term recorded as part of their medical history. One of these patients had CLD and was also identified in the literature search. Another patient had the preferred term *“spleen scan abnormal”* without a diagnosis of lymphoma. These two patients were excluded from this analysis of medical history.

The remaining eight patients were metreleptin-naïve at the time of lymphoma diagnosis. This group comprised six patients with AGL, one patient with CGL, and one patient with an unspecified lipodystrophy type. Although the GSD is a robust system for capturing safety events potentially associated with a suspect medication, medical history data are often incomplete or inconsistently reported. For the eight patients described here, lymphoma was not the primary adverse event that triggered pharmacovigilance reporting. Instead, these patients were entered into the GSD due to other reported adverse events, with lymphoma captured as part of their medical history. Upon review, information related to their lymphoma history was insufficient and possibly contained inconsistent data. Likewise, it was not possible to determine if these patients overlapped the patients identified in the systematic literature review. Due to these limitations, these eight patients are reported separately for the completeness of our analysis, but were excluded from the FAS to maintain data integrity.

## 4 Discussion

The identification of three T-cell lymphomas in three patients with AGL during the clinical program for metreleptin left an open question on whether metreleptin was associated with lymphoma development (30). In the current case series analysis, we identified a greater number of lymphomas in patients with lipodystrophy or CLD who were metreleptin-naïve at the time lymphoma diagnosis (75% of patients) compared with metreleptin-treated patients (25% of patients). While this finding cannot exclude a contributory role for metreleptin in the development of lymphoma, it may suggest that lymphoma development is associated with the underlying disease pathophysiology of lipodystrophy rather than the pharmacological actions of metreleptin. In support of this, the prescribing information for metreleptin in the US and Europe states that a causal relationship between metreleptin and the development or progression of lymphoma has not been established (23–25).

### 4.1 Lymphoma development in patients with AGL

Estimation of the risk of T-cell lymphoma in AGL relative to the general population is challenging due to the rarity of this lipodystrophy type and the lack of formal studies investigating the prevalence of AGL. Analysis of patients with lipodystrophy in the FAS showed that the occurrence of lymphoma was higher in patients with AGL (n=10/15 patients, 67%) compared with other lipodystrophy forms (n=5/15 patients, 33%). Of the 10 patients with AGL, nine had T-cell lymphomas and one had a B-cell lymphoma. This finding is consistent with prior observations reporting a potential relationship between AGL and T-cell lymphoma development (30). However, when the nine patients with AGL and lymphoma in the FAS are considered within the context of the total number of AGL cases reported to date [estimated at less than 200 (5, 7)] and the estimated global prevalence of GL [0.23 cases per million (34)], our data support an increased risk of T-cell lymphoma in patients with AGL relative to the general population (estimated between 1.8 and 4.3 per 100,000) (35, 36). The relationship between AGL and T-cell lymphomas is further evidenced by the predominance of B-cell lymphomas in patients with non-AGL lipodystrophy in the FAS.

Although the etiology of AGL has not been fully elucidated, an association with autoimmune and inflammatory diseases has been reported. Analysis of 79 patients with AGL showed that panniculitis precedes fat loss in approximately 25% of cases, with another 25% of cases associated with autoimmune disease (7). A role for autoimmunity in the pathophysiology of AGL is corroborated by a recent investigation of 40 patients with AGL, among whom, 80% had a clinical history of autoimmune disease and/or autoantibody positivity (5). Notably, autoimmune diseases associated with AGL (e.g., panniculitis, rheumatoid arthritis, Sjögren’s syndrome, systemic lupus erythematosus, and Hashimoto’s thyroiditis) are established risks factor for lymphoma development (5, 7, 37–41). In the FAS, signs of suspected autoimmunity and/or inflammatory disease (e.g., panniculitis, hemophagocytic lymphohistiocytosis, neutropenia, anemia, and leukopenia) were detected in seven of the nine patients with AGL and T-cell lymphoma.

Further interrogation of the FAS revealed that lymphoma development preceded or occurred near to the time of onset of adipose tissue loss in four of the nine patients with AGL and T-cell lymphoma (Patients 5, 7, 9 and 10). This observation supports the view that aberrant lymphoproliferative processes may contribute to adipose tissue loss in AGL. Indeed, it has been hypothesized that the onset of generalized fat loss that followed the lymphoma development in Patient 9 could reflect the development of adipose tissue-directed autoantibodies that occur as a paraneoplastic feature of T-cell lymphomas without direct evidence of the circulating autoantibodies in this publication (42).

Based on these lines of evidence we propose that the occurrence of T-cell lymphoma in patients with AGL may be related to underlying immune dysregulation associated with the development of AGL, characterized by chronic immune activation and inflammation (5, 30). Furthermore, the development of T-cell lymphoma in some patients with AGL may represent a systemic feature of a subset of AGL that is not observed in other lipodystrophy types.

### 4.2 Lymphoma development in CGL and CLD

Leptin deficiency is a key clinical feature of CLD and is frequently reported in patients with lipodystrophy. Previous work has shown that serum leptin levels are severely reduced or undetectable in patients with CGL and CLD (except for ultra-rare cases with biologically inactive or antagonistic leptin), but that these reductions are variable in AGL and less severe in FPLD (10, 14, 15, 17).

Leptin, a pleotropic protein produced largely by adipocytes, is primarily involved in the regulation of energy homeostasis and metabolism, but it also plays an important role in regulating innate and adaptive immunological responses (9, 43–45). Leptin deficiency has been associated with impaired cell-mediated immunity and reduced cytokine responsiveness and may predispose patients with lipodystrophy to infectious disease (46–48). One other report suggests that immune dysregulation increases the risk of lymphoma; however, this has not yet been followed with supportive evidence (49).

Leptin levels were not routinely reported for the patients included in the FAS, and only one patient each with CLD and suspected CGL had lymphomas (Patients 15 and 16) were identified in our analysis (n=2/17, 12% of lymphomas). Hence, our analysis does not provide supporting evidence for an association between leptin deficiency and lymphoma development. Furthermore, to our knowledge, there are no formal scientific studies that demonstrate increased lymphoma risk in leptin-deficient states, including non-AGL lipodystrophy syndromes and CLD.

### 4.3 Lymphoma development in GL-associated atypical progeroid syndrome

One patient (Patient 1) in the FAS had GL-associated APS and experienced two lymphoma events. A role for metreleptin in lymphoma development in this patient cannot be fully excluded; however, EBV infection, post-transplant status, immunosuppressive therapy and chemotherapy, which are established risk factors for lymphoma, were all recorded in this patient (50–53). As such, it is plausible that lymphoma development in this patient is more likely due to post-transplant EBV infection and/or the immunosuppressive therapies administered post-heart transplant surgery rather than metreleptin. Also, while autoimmunity has been documented in individuals heterozygous for the *LMNA* p.(T10I) variant, autoantibodies were not recorded in the medical history of Patient 1 (54).

### 4.4 Study limitations

Our study has several limitations. First, our analysis identified only a small number of patients with lipodystrophy who developed lymphoma. As such, the FAS has a limited sample size from which to draw inferences. Second, it is possible that there are other metreleptin-naïve patients with lipodystrophy reported in other literature databases not searched by us and thus omitted from the FAS. Third, our analysis and interpretations are limited to the amount of clinical information reported for each case. For example, little information, if any, was provided for the leptin levels in each patient, hence the role of leptin deficiency in lymphoma development cannot be fully explored here. Fourth, the prevalence of AGL has not been formally investigated. Therefore, our assessment of the risk of T-cell lymphoma in patients with AGL relative to the general population is based on earlier studies regarding the prevalence of GL and an estimated number of AGL cases from the literature. Fifth, while we present some possible explanations for the association between lymphoma and AGL, to our knowledge, no formal scientific studies exploring the molecular basis of the possible association between these two diseases have been conducted. Such studies could provide novel insight into the complex relationship between immunoproliferative disorders of lymphocytes, autoimmune processes, and the onset of adipose tissue loss. 

## 5 Conclusions

In conclusion, our analysis supports the hypothesis that patients with lipodystrophy syndromes, especially AGL, may have an elevated risk of developing lymphomas. Furthermore, our analysis suggests that this increased risk is more likely associated with underlying disease processes rather than the pharmacological effects of metreleptin. These disease processes may be broad and multisystemic and not only related to presence of lipodystrophy but also to development of other disease manifestations. Further work is needed to fully explore the pathological mechanisms that potentially link immunoproliferative disorders, mediated by lymphocytes (and potentially other immune cells), and the lipodystrophy phenotype.

## Data Availability

The original contributions presented in the study are included in the article/**Supplementary Material**. Further inquiries can be directed to the corresponding author.
