# Supplementary material for: A real-world pharmacovigilance assessment and literature review of lymphoma development in lipodystrophy
Source: Front Endocrinol (Lausanne). 2025 May 21;16:1582715. doi: 10.3389/fendo.2025.1582715 (PMC12133509; doi:10.3389/fendo.2025.1582715)
Supplement: Supplementary file 1 [file DataSheet1.docx]

*Supplementary Material*

A Real-World Pharmacovigilance Assessment and Literature Review of Lymphoma Development in Lipodystrophy and Congenital Leptin Deficiency

Synopsis of the clinical cases presented in the final analysis set (FAS)

**1. Lymphomas in metreleptin-treated patients**

- 1. B-cell and brain lymphomas in a metreleptin-treated patient

Patient 1 (male) was identified through analysis of the global safety database (GSD) of the marketing authorization holder (MAH) for metreleptin. He was previously described prior to lymphoma development in a real-world study of metreleptin in a Spanish cohort (1, 2). Follow-up pharmacovigilance data for this patient were collected by the MAH for metreleptin following the development of lymphomas since this earlier publication.

Patient 1 was noted to have fat loss beginning at about 3.5 years of age followed by a diagnosis of diabetes mellitus, hypertriglyceridemia, hepatic steatosis and dilated cardiomyopathy at age 8 years. Generalized lipodystrophy-associated atypical progeroid syndrome (caused by a *LMNA* c.29C>T (p.T10I) pathogenic variant) was confirmed at age 8 years (1, 2). Metreleptin was initiated (0.06 mg/kg/day) and achieved metabolic control. However, due to worsening heart failure, the patient received a heart transplant (age 13 years) followed by immunosuppressant therapy (1).

At approximately 19 years of age, the patient was diagnosed with post-transplant polymorphic myeloproliferative syndrome, characterized by B-lymphoid population and Epstein-Barr virus (EBV) positivity and was treated with chemotherapy. At age 21 years the patient presented with headache, vomiting, and decreased consciousness. Computed tomography (CT) scans revealed severe obstructive hydrocephalus and cerebellar abnormalities suggestive of infiltrative lesion/edema. These findings were attributed to the previous lymphoma. The patient succumbed to multi-organ failure at age 21 years. Lymphoma development in this patient was assessed as not related to metreleptin treatment.

- 1. T-cell lymphoma in metreleptin-treated patients

Three patients with acquired generalized lipodystrophy (AGL) and T-cell lymphoma were reported by Brown et al. (2016) (3).

Patient 2 (female) developed AGL between 1 and 2 years of age (3). Evaluation at the US National Institute of Health (NIH) at age 11 years revealed hypertriglyceridemia, severe insulin resistance, and metabolic dysfunction-associated steatohepatitis (MASH) with possible cirrhosis. No hematologic abnormalities were detected, and autoimmune markers were negative. Metabolic tests one-year post-metreleptin initiation showed stable glycated hemoglobin (HbA1c), improved triglyceride levels and reduced insulin levels. After 21 months of metreleptin treatment, biopsy of a non-tender, 5×4 cm mass from the patient’s right breast revealed anaplastic lymphoma kinase (ALK)-positive anaplastic large cell lymphoma with a clonal T-cell population. Metreleptin was discontinued. Tests conducted one month after the excisional biopsy revealed that the patient’s metabolic disease had worsened, with increases in fasting insulin, glucose, triglycerides, and elevated alanine transaminase (ALT) and aspartate transaminase (AST) levels. As the risk of worsening metabolic disease was considered greater than the risk posed by metreleptin with respect to lymphoma development, metreleptin was reinitiated six weeks after biopsy. No residual lymphoma was detected after nine months of chemotherapy.

Patient 3 (female) developed fat loss consistent with AGL, diabetes mellitus, hypertriglyceridemia and hepatic steatosis in the fifth decade of life (3). Neutropenia occurred at 58 years of age and granulocyte colony stimulating factor (GCSF) treatment was started. A subsequent bone marrow biopsy revealed atypical T-cell lymphocytosis and myeloid maturation. Her initial evaluation at the NIH (at age 59 years) showed that most hematological makers were normal on GCSF except for an elevated lymphocyte count. Her autoimmune markers were negative. Metreleptin treatment was initiated. Four months later, multifocal intraductal carcinoma of the right breast without lymph node invasion was detected and the patient underwent mastectomy. Eight months post-metreleptin treatment, she developed nodular skin plaques; skin and bone marrow biopsies revealed peripheral T-cell lymphoma (PTCL). Metreleptin was discontinued. The patient died from PTCL eight months after lymphoma diagnosis.

Patient 4 (male) developed generalized loss of subcutaneous fat (starting at age 63 years) over several months (3). Initial evaluation at the NIH (conducted 4 years later) showed that he had elevated levels of HbA1c, fasting plasma glucose, triglycerides, ALT and AST, and leukopenia and neutropenia. A liver biopsy revealed steatohepatitis. Physical examination identified diffuse lymphadenopathy and a 2–3 cm purplish skin lesion on a lower extremity. Autoimmune markers were negative at this time. Metreleptin and GCSF treatment for neutropenia were initiated, and metabolic parameters improved after 8 months. Worsening of skin lesions over a 30-day period was reported by the patient around this time and biopsy of the lesions on the right leg showed PTCL. Metreleptin treatment was discontinued. The patient died from PTCL 6 months after diagnosis.

1. Lymphomas in metreleptin-naïve patients

In total, 12 lymphoma events were identified in 12 metreleptin-naïve patients in the FAS. T-cell lymphomas were reported in two patients with AGL (Patients 5 and 10), and one B-cell lymphoma was identified in one patient with congenital leptin deficiency (CLD, Patient 16) who were metreleptin-naïve at the time of lymphoma diagnosis but who later received metreleptin. Nine events occurred in nine patients who never receive metreleptin (**Table 3** in the main manuscript). A synopsis of each case is detailed below.

- 1. T-cell lymphomas in metreleptin-naïve patients

Six T-cell lymphomas were identified in six metreleptin-naïve patients, all with AGL, in the FAS.

Patient 5 (female) was diagnosed with poikilodermatous mycosis fungoides (a type of primary cutaneous T-cell lymphoma) at age 39 years that subsequently resolved (3). Over 25 years later, at age 60 years, she presented with rapid generalized fat loss, hypertriglyceridemia and diabetes mellitus and low leptin levels after a flu-like illness. At age 68 years, she developed anemia and presented with an increased abdominal girth due to ascites containing adenocarcinoma cells of undetermined origin. She received metreleptin for a total of 2.6 months for the treatment of AGL under an expanded access program and recorded both improved triglycerides and HbA1c levels. During metreleptin treatment, there was no evidence of mycosis fungoides recurrence. The patient subsequently decided to discontinue metreleptin and declined treatment for the adenocarcinoma. She died from adenocarcinoma soon thereafter (3).

Patient 6 (male) presented at age 32 years with recurrent fevers, chills and fatigue, and accompanying subcutaneous flesh-colored nodules diagnosed as lympho-histiocytic panniculitis (4). Thereafter, the patient reported diffuse loss of fat on his face, trunk, and extremities consistent with AGL. At age 45 years, laboratory analysis showed leukopenia, hypertriglyceridemia, and elevated levels of ALT, AST, and creatine kinase. Around this time, he developed PTCL and was started on chemotherapy, which stabilized the lipoatrophy and decreased the size and frequency of the subcutaneous nodules (4).

Patient 7 (male) was diagnosed with PTCL (at age 46 years) following the development of fever, malaise, night sweats, weight loss, edema, and lymphadenopathy three years earlier (5). He failed to respond to two different chemotherapy regimens. Eighteen months after lymphoma diagnosis (at age 47–48 years), he developed fat loss from the face, abdomen and limbs. This was accompanied by the appearance of prominent veins and musculature, hepatomegaly with fatty infiltration, and a metabolic profile consistent with AGL (e.g., type 2 diabetes, severe insulin resistance, hyperphagia, hypertriglyceridemia). Although the patient had no preceding history of panniculitis or autoimmunity, it was concluded that fat loss was caused by an overlap of autoimmune-related (Type 2) and idiopathic varieties of AGL (Type 3) (5).

Patient 8 (a 33-year-old female) underwent chemotherapy and an allogenic stem cell transplant for treatment of a subcutaneous panniculitis-like T-cell lymphoma (SPTCL) complicated by hemophagocytic lymphohistiocytosis (HLH) at age 26–27 years (6). During the seven years after treatment, her T-cell lymphoma remained in remission, but she developed generalized adipose tissue loss and a metabolic profile consistent with AGL (e.g., hypertriglyceridemia, elevated HbA1c and plasma glucose, and proteinuria). Inspection of the patient’s medical history including photographic evidence, determined that the onset of adipose tissue loss preceded the patient’s T-cell lymphoma diagnosis (6).

Patient 9 (a 52-year-old African-American male with AGL) was diagnosed with SPTCL at age 34 years that resolved spontaneously without any treatment (7). At approximately 46 years of age, he presented with new nodules diagnosed as either SPTCL or lupus panniculitis. Shortly thereafter, symptoms (e.g., fever, weight loss, night sweats, generalized edema, and cytopenia) consistent with HLH developed. He received immunosuppressant therapy followed by allogeneic hematopoietic stem cell transplantation. Generalized subcutaneous fat loss affecting the trunk, face, and upper and lower extremities was evident and persisted after HLH treatment. The patient’s most recent metabolic evaluation showed normal levels of total cholesterol, high-density lipoprotein (HDL)-cholesterol, triglycerides, fasting glucose level and leptin, but slightly elevated HbA1c (7).

Patient 10 (a 68-year-old female) was diagnosed with left inguinal angioimmunoblastic T-cell lymphoma (AITL) at age 58 years (8). After completing six rounds of chemotherapy (cyclophosphamide, hydroxydaunorubicin, oncovin, and prednisone regimen) she had a relapse in the right inguinal and received five cycles of rituximab, ifosfamide, carboplatin, and etoposide phosphate (R-ICE regimen) leading to complete remission. At age 64 years, she developed generalized fat loss and prominent musculature. Glucose-lowering therapy (including insulin) was initiated for the treatment of diabetes; however, this resulted in inadequate glycemic control. Based on these clinical features, AGL was diagnosed. Metreleptin was introduced at age 68 years leading to a normalization of blood glucose levels and cessation of insulin therapy.

- 1. B-cell lymphomas in metreleptin-naïve patients

Three B-cell lymphoma events were identified in three metreleptin-naïve patients with lipodystrophy in the FAS.

Patient 11 (a 16-year-old male) was referred for diabetes, acanthosis nigricans, and hepatomegaly with elevated liver enzymes (3). AGL was diagnosed (at approximately 10 years of age) after the development of lipoatrophy following a pulmonary infection (genetic tests for CGL were negative). Physical examination revealed splenomegaly and CT scans showed mediastinal and mesenteric lymphadenopathy. Further assessment identified anemia and elevated levels of C-reactive protein and fibrinogen (indicative of inflammation) and culminated in a diagnosis of Burkitt lymphoma. Remission of lymphoma was achieved after chemotherapy, but lipoatrophy and diabetes remained unchanged after 18 months of follow-up (3).

Patient 12 (a 40-year-old female) first recognized increased musculature in her limbs and a concomitant loss of fat from her face around puberty (9). She was diagnosed with polycystic ovarian syndrome in adolescence and at 29 years of age experienced an episode of acute pancreatitis caused by severe hypertriglyceridemia. Approximately one-year later she was diagnosed with gestational diabetes mellitus after becoming pregnant and received insulin. Upon presentation, reduced levels of fat were evident in her face, limbs, and trunk. A whole-body magnetic resonance image showed a near-total loss of fat with hepatic steatosis suggestive of generalized lipodystrophy. However, she also had well-preserved fat in the mons pubis and external genital region more consistent with partial lipodystrophy. Genetic testing confirmed a pathogenic heterozygous variant (p.(K486E); c.1456A>G) in the *LMNA* gene, a gene which is usually associated with familial partial lipodystrophy (FPLD). At age 40, a CT scan revealed lymphadenopathy in multiple lymph nodes and biopsy was diagnostic for low-grade B-cell follicular lymphoma. Laboratory data collected prior to the initiation of chemotherapy showed normal ALT, AST, insulin and HbA1c levels but increased triglyceride, total cholesterol and low-density lipoprotein levels (9).

Patient 13 (an adult female) exhibited loss of subcutaneous fat deposits in the forearm, lower limbs, and buttocks, with prominent musculature of the lower limbs at age 48 years (some of these phenotypic features were noticed by the patient at age 12 years) (10). Excess fat deposition around the face, neck, and trunk was also evident. The patient had no history of autoimmune or infectious diseases. Laboratory evaluation showed increased HbA1c and triglycerides and total cholesterol but no other abnormal endocrine findings. Genetic tests for FPLD were negative but a review of the patient’s family history and fat distribution patterns led to a diagnosis of FPLD. CT scans showed an enlarged para-aortic lymph node and polyps in the terminal ileum were detected by colonoscopy. A diagnosis of intestinal follicular B-cell lymphoma was confirmed by biopsy of a lymphoid follicle taken from the ileal mucosa (10).

- 1. Hodgkin lymphomas in metreleptin-naïve cases

Three cases of Hodgkin lymphoma were identified in three metreleptin-naïve cases in the FAS.

Patient 14 (a 42-year-old female) was diagnosed with juvenile dermatomyositis, a rare autoimmune disease that causes muscle inflammation and degeneration, at age 3 years (11). The patient responded poorly to long-term immunosuppressive therapy, which was stopped at age 21 years. At age 31 years, she was diagnosed with nodular-sclerosis classical Hodgkin lymphoma and achieved full remission following chemotherapy. Seven years later (age 38 years), she presented with several clinical complications including menstrual irregularities, hirsutism, and generalized muscle atrophy. A distended abdomen (with hepatosplenomegaly) and pronounced lipodystrophy of the face and limbs became apparent, and evidence of autoimmunity (e.g., positivity for anti-transcriptional intermediary factor gamma protein antibody) was detected. Metabolic assessment showed normal fasting plasma glucose and liver transaminases levels, but elevated insulin and triglyceride levels (11).

Patient 15 (a 22-year-old female) showed generalized absence of fat consistent with GL at the age of 3 months (12). One month later, subcutaneous nodules developed on the patient’s extremities. Skin biopsies at the age of 8 years were consistent with scleroderma. Later, during adulthood, biopsy of the left supraclavicular lymph node resulted in a diagnosis of Hodgkin lymphoma (12). Although not confirmed in the source clinical report, this patient likely had CGL given the age at which fat loss occurred.

Patient 16 (female) was diagnosed with CLD after referral to a US medical center at age 20 years. Hodgkin lymphoma was identified following an incidental finding of axillary lymphadenopathy (13, 14). She had a history of hyperphagia and weight gain (evident from 3 months of age) and comorbidities of hydrocephalus, developmental delay, hyponatremia, autoimmune thyroid disease, growth hormone deficiency and prediabetes. Serum leptin levels were undetectable. Genetic analysis revealed that the patient was homozygous for the pathogenic variant c398delG in exon 3 of the leptin gene, causing a frameshift/premature stop codon. Clinical remission of the Hodgkin lymphoma was achieved via a combination of chemotherapy and radiotherapy. Thereafter, she started off-label metreleptin treatment (13, 14).

**References**

1. Araujo-Vilar D, Sanchez-Iglesias S, Guillin-Amarelle C, Castro A, Lage M, Pazos M, et al. Recombinant Human Leptin Treatment in Genetic Lipodystrophic Syndromes: The Long-Term Spanish Experience. *Endocrine* (2015) 49(1):139-47. Epub 20141104. doi: 10.1007/s12020-014-0450-4.

2. Hussain I, Patni N, Ueda M, Sorkina E, Valerio CM, Cochran E, et al. A Novel Generalized Lipodystrophy-Associated Progeroid Syndrome Due to Recurrent Heterozygous LMNA P.T10i Mutation. *J Clin Endocrinol Metab* (2018) 103(3):1005-14. doi: 10.1210/jc.2017-02078.

3. Brown RJ, Chan JL, Jaffe ES, Cochran E, DePaoli AM, Gautier JF, et al. Lymphoma in Acquired Generalized Lipodystrophy. *Leuk Lymphoma* (2016) 57(1):45-50. Epub 20150512. doi: 10.3109/10428194.2015.1040015.

4. Yiannias JA, DiCaudo DJ, Maskin E. Peripheral T-Cell Lymphoma Presenting as Lipoatrophy and Nodules. *Int J Dermatol* (2006) 45(12):1415-9. doi: 10.1111/j.1365-4632.2006.02888.x.

5. Aslam A, Savage DB, Coulson IH. Acquired Generalized Lipodystrophy Associated with Peripheral T Cell Lymphoma with Cutaneous Infiltration. *Int J Dermatol* (2015) 54(7):827-9. Epub 20131029. doi: 10.1111/ijd.12185.

6. Esfandiari NH, Rubenfire M, Neidert AH, Hench R, Eldin AJ, Meral R, et al. Diagnosis of Acquired Generalized Lipodystrophy in a Single Patient with T-Cell Lymphoma and No Exposure to Metreleptin. *Clin Diabetes Endocrinol* (2019) 5:4. Epub 20190314. doi: 10.1186/s40842-019-0076-9.

7. Hoff FW, Xing C, Garg A. A Novel Subtype of Acquired Generalized Lipodystrophy Associated with Subcutaneous Panniculitis-Like T-Cell Lymphoma. *JCEM Case Rep* (2024) 2(5):luae069. Epub 20240427. doi: 10.1210/jcemcr/luae069.

8. Ebihara K, Murakami A, Kasuya Y, Ebihara C, Isoda M. Efficacy of Leptin Therapy in a Patient with Acquired Generalized Lipodystrophy Whose Etiology Might Be Common for T-Cell Lymphoma. *Endocrine Society Annual Meeting 2018*; Chicago, IL, USA. Conference abstract in: Endocrine Reviews. Volume 39, Issue Supplement. 1st April 2018.

9. de Andrade NXS, Adiyaman SC, Yuksel BD, Ferrari CT, Eldin AJ, Saydam BO, et al. Unusual Presentations of Lmna-Associated Lipodystrophy with Complex Phenotypes and Generalized Fat Loss: When the Genetic Diagnosis Uncovers Novel Features. *AACE Clin Case Rep* (2020) 6(2):e79-e85. Epub 20200304. doi: 10.4158/ACCR-2019-0366.

10. Iwanishi M, Ebihara K, Kusakabe T, Washiyama M, Ito-Kobayashi J, Nakamura F, et al. Primary Intestinal Follicular Lymphoma and Premature Atherosclerosis in a Japanese Diabetic Patient with Atypical Familial Partial Lipodystrophy. *Intern Med* (2014) 53(8):851-8. Epub 20140415. doi: 10.2169/internalmedicine.53.1713.

11. Rego de Figueiredo I, Guerreiro Castro S, Bernardino V, Silva Nunes J, Alves P, Moraes-Fontes MF. Juvenile Dermatomyositis Forty Years On: Case Report. *Neuromuscul Disord* (2018) 28(9):791-7. Epub 20180630. doi: 10.1016/j.nmd.2018.06.011.

12. Hall SW, Gillespie JJ, Tenczynski TF. Generalized Lipodystrophy, Scleroderma, and Hodgkin's Disease. *Arch Intern Med* (1978) 138(8):1303-4.

13. Torchen L, Hakamy B, Gordon L, Yaseen N, Neff L. Psun125 Lymphoma in Congenital Leptin Deficiency: Comorbidity or Adverse Effect? .

Journal of the Endocrine Society, Volume 6, Issue Supplement_1, November-December 2022, Page A31, https://doi.org/10.1210/jendso/bvac150.065

14. Torchen LC, Hakamy B, Gordon LI, Marsh EE, Yaseen NR, Neff LM. OR33-03: Congenital Leptin Deficiency: Clinical Insights from the First Reported US Cases.

*Journal of the Endocrine Society, Volume 4, Issue Supplement_1, April-May 2020, OR33–03, https://doi.org/10.1210/jendso/bvaa046.1059*
